# Climate change anxiety in the scientific community: an exploratory study with Chilean climate change-related scholars

**DOI:** 10.3389/fpsyg.2025.1507487

**Published:** 2025-08-13

**Authors:** Rodolfo Sapiains, Gabriela Azócar, Gonzalo Palomo-Vélez, Roberto Rondanelli

**Affiliations:** ^1^Center for Climate and Resilience Research, University of Chile, Santiago, Chile; ^2^Facultad de Ciencias, Sociales Universidad de Chile, Santiago, Chile; ^3^Instituto de Ciencias Sociales, Universidad de O’Higgings, Rancagua, Chile; ^4^Departamento de Geofísica, University of Chile, Santiago, Chile

**Keywords:** climate anxiety, ecoanxiety, climate change, Chile, mental health

## Abstract

**Background:**

Eco-anxiety or climate change anxiety can be defined as a chronic fear of environmental doom that for some people might trigger clinical psychological issues. Although the study of this phenomenon is growing, there is not much understanding of the psychological consequences that studying climate change can have on scholars who are overexposed to information that is generally full of negative projections. This study aims at exploring to what extent continued exposure to scientific information about climate change affects those who research it.

**Methods:**

We conducted an online survey with a sample of climate scientists from Chile (*n* = 249), one of the most vulnerable countries to climate change. A Spanish-translated and adapted version of Clayton and Karazsia’s climate change anxiety scale was used along with single items to assess self-reported climate change anxiety, and sociodemographic factors.

**Results:**

Most Chilean climate change scientists are being emotionally affected by climate change. However, high levels of self-reported ecoanxiety contrast with more moderate results when measuring ecoanxiety as a whole and in both subscales, cognitive-emotional and functional. Women, young people, and those who do not have children, express more emotional and functional impacts. Social scientists showed higher climate change anxiety levels than natural scientists.

**Conclusion:**

Although for most participants climate change anxiety is not affecting life functioning, this does not necessarily mean that it will not affect them in the future. We believe that research centres and teams must develop strategies to help scholars cope with the psychological consequences of working on climate change.

## 1 Introduction

Climate change is having a growing impact on people’s mental health (Hayes et al., 2018; Maibach et al., 2021; Shahid et al., 2021). This can be related to experiencing extreme weather events, new adverse climatic conditions, negative perspectives about the future of the planet or a high exposure to catastrophic climate change information (Palinkas et al., 2020). Different concepts have been proposed to describe this issue such as ecological grief (Cunsolo and Ellis, 2018), solastalgia (Galway et al., 2019), eco-depression, eco-guilt, and ecoanxiety (Ágoston et al., 2022; Coffey et al., 2021). The latter has been defined as a chronic fear of environmental doom, that for some people might trigger clinical psychological issues. A metanalysis of ecoanxiety studies (Boluda-Verdú et al., 2022) found associations with multiple psychological symptoms including depression, stress, functional impairment, insomnia, and anxiety itself.

Climate anxiety specifically refers to the emotional responses people express to the anticipated impacts of climate change. Although the extent these emotional responses are pathological or non-pathological varies in the literature (Van Valkengoed, 2023; van Valkengoed and Steg, 2023), it is accepted that high levels of ecoanxiety, or climate change anxiety can have negative impacts on people’s wellbeing and in different life dimensions, which might be expressed in difficulties to sleep, lack of concentration at work or school, problems to enjoy social activities, among others (McBride et al., 2021). At the same time, new studies are showing levels of climate change anxiety are influenced by factors such as sex, with women generally being more concerned about climate change and environmental issues (Calixto, 2019; du Bray et al., 2019; Sapiains et al., 2023); age, with younger generations generally more psychologically affected by climate change and the state of the environment (Hickman et al., 2021; Vaznonienė, 2023); and having or not having children, without conclusive results but with some trends indicating that the former are generally more concerned as they worry for the future of their children.

In this context, there is a group of people who, due to their constant exposure to climate information and deep understanding of future projections of climate change, might be significantly more affected than the general population: climate change scholars. Currently there is not much understanding of the psychological impacts that studying climate change can have on researchers who are continuously exposed to information, that many times is full of negative projections, and who generally have a high sense of responsibility to provide climate change evidence to support climate action (Getson et al., 2021) but with a lack of efficacy to influence climate policies to address mitigation and adaptation challenges (Latter et al., 2024). In cases like Chile, it is also likely to have been affected by severe climatic phenomena in the last decades (e.g., megadrought in the central zone, massive fires, floods), mounting pressure on Chilean scholars as direct experience of climate change impacts is one of the main triggers of mental health-related issues (Cunsolo and Ellis, 2018; Drew, 2013; Krüger et al., 2022; Young et al., 2020). Thus, it is likely that many Chilean climate change scientists can be emotionally affected by climate change.

This article reports a study aimed at exploring to what extent continued exposure to scientific information about climate change affects those who research it. We conducted an online survey with a sample of climate scientists from Chile, a country highly vulnerable to climate change impacts, meeting seven of the nine vulnerability criteria defined by the United Nations Framework Convention on Climate Change [UNFCCC] (Oficina de cambio climático, 2014). We measure the influence of age, gender, years working on climate change, and disciplines on climate change anxiety in this group of scientists. We expect similar results to the ones observed in the general population regarding sex, age, and having children, but we are particularly interested in exploring the effect of two less observed factors: disciplines and years working on climate change. While the impact of experience may be associated with researchers’ age, the discipline variable might reflect inherent differences between natural sciences and social sciences. These differences are likely to affect perceptions of climate change, considering that social sciences historically began addressing the topic later than natural sciences whereby the maturity and development levels of these fields vary significantly. Additionally, their research approaches—such as objectives, methods, and focus areas—tend to differ considerably. Moreover, in many cases natural scientists are more exposed to the natural environment which might work as a protective factor (Li et al., 2025). To our knowledge there are not climate anxiety studies integrating these disciplinary differences. Thus, considering the state of the art on this issue, and our knowledge about the Chilean climate science community, we proposed the following hypothesis:

*H1*: Scientists, as they are continuously exposed to climate change information, show relatively high levels of climate anxiety (i.e., above of the theoretical midpoint of the scale).

*H2*: Climate change anxiety is higher in female scholars than in male scholars.

*H3*: Scholars with children express higher levels of climate anxiety than scholars without children.

*H4*: Climate change anxiety decreases with the age of the scientists.

*H5*: Climate change anxiety is higher in social scientists than in natural scientists.

*H6*: Climate change anxiety decreases with years dedicated to study climate change.

## 2 Materials and methods

### 2.1 Participants

We recruited participants through email invitations sent to different Chilean-based research institutes, centres, and groups that investigate climate change (e.g., Center for Climate and Resilience Research, www.cr2.cl; Transdisciplinary Systemic Studies Center, www.nest-r3.cl; Austral Science NODE; www.nodocienciaaustral.cl; CITRID, www.citrid.uchile.cl). Further, invitations were also directed to relevant academics and faculties of Chilean Universities, as well as, to public and private institutions that work and do research on climate change and environmental issues in the country (Global Methane Hub, www.globalmethanehub.org; Ministry of the Environment; https://www.gob.cl/en/ministries/ministry-of-the-environment). In total, 382 people started the online survey, of whom 319 answered the measurements related to climate anxiety. However, 70 participants indicated they did not work in climate change-related research (e.g., journalists, for instance, who worked on climate change communication but did not research it). As such, the final sample included 249 participants (Table 1), living mostly in central and south regions of the country. Particularly, most participants lived in the metropolitan region (59.4%), followed by the Bio-Bio region (6.8%), the Valparaiso region (6.0%), and the Maule region (5.2%) (Table 1).

**Table 1:** Characteristics of the sample.

Variables		Count	%
Gender^1^	Male	105	42.2%
	Female	139	55.8%
	Other or prefer not to answer	5	2.0%
Age Groups	20 to 35 years	111	44.6%
	36 to 45 years	88	35.3%
	46 years and older	50	20.1%
Do you have children?	No	154	61.8%
	Yes	95	38.2%
Science field	Social sciences	105	42.2%
	Natural sciences	144	57.8%

### 2.2 Procedure

The questionnaire began with a short explanation of the research goals. Then, a brief definition of climate change anxiety was presented, describing it as the emotional distress that a person can experience in response to the anticipated impacts of climate change. After that, participants were asked how often they experience this kind of emotional distress due to climate change (1 = never to 5 = every day). Next, they were invited to answer a series of questions taken from the climate anxiety scale created by Clayton and Karazsia (2020) and a measurement of general anxiety (Spitzer et al., 2006). The order of the items associated to each scale was randomized. Afterward, participants were asked to indicate which they thought was the main cause of climate change and provide some demographics (e.g., age, gender, if they had children, and where they lived) as well as relevant information regarding their scientific work on climate change (e.g., how many years have they been working on climate change research?; and their primary research area). Finally, participants were thanked for participating.

### 2.3 Measurements

#### 2.3.1 Climate change anxiety

People’s emotional distress due to climate change was measured using a Spanish-translated and adapted version of Clayton and Karazsia’s climate change anxiety scale. The original scale consists of 22 five-point Likert-type items (1 = never to 5 = always) that reflect four subscales. Two of these subscales constitute core dimensions of climate change anxiety: cognitive and emotional impairment (e.g., “Thinking about climate change impairs my ability to concentrate”) and functional impairment (e.g., “My concerns about climate change make it hard for me to have fun with my family or friends”). The other two subscales evaluate correlates of climate change anxiety, particularly, personal experience with climate change (e.g., “Climate change affects me directly”) and behavioral engagement. To streamline the administration of a concise questionnaire, two items were not assessed in our adapted version of the scale: one from the functional impairment dimension (“My concerns about climate change undermine my ability to work to my potential”) and another from the experiential dimension (“I have known someone who has been directly affected by climate change”). These items were deemed conceptually too similar to other items of the scale. Furthermore, the behavioral engagement subscale, was omitted from the current investigation owing to its exploratory nature. As such, our adapted version of the climate change anxiety scale consisted of 14 items measuring cognitive-emotional, and functional impairment, as well as experience with climate change. In the current study, each measured subscale showed adequate internal consistency (*α* cognitive-emotional impairment = 0.82; α functional impairment = 0.74; α functional experience = 0.75; see Annex 1 for the verbatim text of the items).

#### 2.3.2 Generalized anxiety

We measured generalized anxiety using a Spanish-translated version of the Generalized Anxiety Disorder Scale (Spitzer et al., 2006). This scale identifies probable cases of generalized anxiety disorder based on the DSM-5 criteria. The GAD-7 consists of 7 four-point Likert-type items that ask about the frequency of different anxiety-related symptoms (e.g., “Not being able to stop or control worrying,” “Becoming easily annoyed or irritable”) over the last 2 weeks (1 = never to 4 = nearly every day). The GAD-7 showed adequate internal consistency in this application (*α* = 0.87).

### 2.4 Analysis approach

Analyses were carried out focusing on both the overall scores of climate change anxiety and its core dimensions separately (cognitive-emotional and functional impairment). First, descriptive statistics and central tendency measures were used to evaluate the level of climate change anxiety reported by our sample. Then, the associations (i.e., Pearson correlation coefficients) between climate change anxiety and relevant correlates (i.e., self-reported climate anxiety) were explored to provide further evidence for the construct’s validity and test predictions related to its overlap with general anxiety. After this, a series of single analyses of variance (ANOVA) were carried out to examine whether overall scores of climate change anxiety varied depending on participants’ gender (male vs. female),^1^ parent status (yes vs. no) science field (social sciences vs. natural sciences), and the amount of time dedicated to studying climate change (less than 2 years, 2–5 years, 5–10 years, or more than 10 years). Further, to explore whether the cognitive-emotional and functional impairment subscales varied depending on the mentioned factors, a series of multivariate analyses of variance (MANOVA) were conducted. MANOVAs were preferred over separate ANOVAs for analysing these subscales because they were correlated (*r* = 0.73, *p* < 0.001). The Pillai’s V statistic was used to determine whether there were significant differences between groups. Pillai’s V statistic is one of the most robust statics when it comes to unbalanced grouping factors and potential violations of assumptions (Meyers et al., 2017). Finally, to explore whether climate change anxiety decreases with the age of the scientists, the associations between age, overall scores of climate change anxiety, and scores on each core dimension were examined. All analyses were conducted using IBM SPSS Statistics, Version 29.

### 2.5 Ethical considerations

We adhered to all ethical standards for this type of research, as it involved an online survey that posed no risks to participants. After providing a summary of the study, including its goals and methods, participants were asked to give informed consent before proceeding to complete the survey. They were informed about the expected length of the questionnaire, provided with the researchers’ contact information, and assured that participation was voluntary and anonymous. Participants were also informed that they had the right to withdraw from the study at any time without facing any penalties. Upon completion of the study, results will be shared with the institutions, organizations, and scientists who collaborated on the research. They will also be invited to participate in dissemination activities where the findings will be presented.

## 3 Results

### 3.1 How common is climate change anxiety?

As previously described, we began by providing the definition of climate change anxiety to our respondents and asked them to self-report their levels of climate change distress on a scale from 1 to 5. The mean and standard deviation results showed relatively high scores, that is, above the midpoint of the scale (*M* = 3.36, SD = 0.97). Most participants indicated experiencing emotional distress due to climate change “frequently” (36.9%) and “sometimes” (36.5%), followed by “rarely” (11.6%), “every day” (10%), and “never” (4.8%). In contrast, the results of the Clayton and Karazsia scale, when measured in its entirety, exhibited a moderate score below the midpoint of the scale (*M* = 1.94, SD = 0.61), which also holds true for the cognitive-emotional (*M* = 1.84, SD = 0.62) and functional (*M* = 2.14, SD = 0.72) subscales separately. Thus, the results indicate that H1 is only partially supported.

When focusing on Clayton and Karazsia’s scale at the item-level frequencies, the results shown in Table 2 reveal significant insights into the cognitive-emotional impacts of climate change on participants. A substantial portion of participants never or almost never experience cognitive and emotional difficulties related to climate change. Specifically, 50.2% never have trouble sleeping due to climate change, and 65.5% never have nightmares about it. However, some participants do face these issues: 4.0% almost always or always find it hard to concentrate, and 10.8% almost always or always feel like crying when thinking about climate change. Additionally, 10% almost always or always feel they cannot manage their emotions regarding climate change.

**Table 2:** Items subscale cognitive-emotional.

Response alternatives	Items							
	Thinking about climate change makes it difficult for me to concentrate	Thinking about climate change makes me want to cry	Thinking about climate change makes it difficult for me to sleep	I have nightmares about climate change	I feel like I can’t manage my emotions regarding climate change	I question myself why I feel so bad about climate change.	I write down my thoughts about climate change and analyze them	I question myself why I react so badly to news about climate change
Never	35,3%	39,8%	50,2%	65,5%	39,8%	47,0%	59,8%	45,4%
Almost never	34,9%	23,3%	30,1%	19,7%	31,7%	27,7%	14,9%	30,5%
Sometimes	25,7%	26,1%	17,7%	12,9%	18,5%	20,1%	20,9%	18,5%
Almost always or always	4,0%	10,8%	2,0%	2,0%	10,0%	5,2%	4,4%	5,6%
Total	100%	100%	100%	100%	100%	100%	100%	100%

In the functional impairment dimension, the effects of anxiety mainly manifest in overthinking about climate change, a phenomenon frequently observed in 45.4% of cases. Second is the ability to balance concern about climate change with the needs of the family (35.7%). Less frequently, concern about climate change affects the ability to have fun with the family (34.5%) and to work or study (30.5%) (Table 3).

**Table 3:** Items subscale functional impairment.

Response alternatives	Items			
	My concerns about climate change make it hard for me to have fun with my family or friends	I have problems balancing my concerns about climate change with the needs of my family	My concerns about climate change interfere negatively with my ability to get work or school assignments done	My friends say I think about climate change too much
Never	27,3%	30,1%	34,9%	33,3%
Almost never	38,2%	34,1%	34,5%	21,3%
Sometimes	29,7%	29,7%	26,9%	27,3%
Almost always or always	4,8%	6,0%	3,6%	18,1%
Total	100%	100%	100%	100%

### 3.2 How does climate change anxiety relate to experiencing climate change and anxiety symptoms?

The criterion validity of the climate change anxiety scale, as well as its two constituting subscales, was evaluated by examining their relationship with a generalized anxiety scale as well as the self-reported measure of climate change anxiety. The climate change anxiety scale and both subscales, cognitive-emotional and functional impairment, demonstrated moderate-to-strong associations with self-reported climate change anxiety (0.66, 0.62 and 0.61, respectively). Lastly, the associations between the climate change anxiety scale and its subscales (cognitive-emotional and functional impairment) with generalized anxiety revealed that, although both types of anxiety are distinguishable, they still exhibit a partial overlap, with positive moderate correlations between each of them (0.47, 0.44 and 0.43 respectively) (Table 4).

**Table 4:** Associations between the climate anxiety scale, its constituting factors, experiencing climate change, self-reported climate anxiety, and generalized anxiety.

	Construct	1	2	3	4	5
1	Climate anxiety overall scale	-	0.96**	0.88**	0.66**	0.47**
2	Cognitive-emotional impairment		-	0.73**	0.62**	0.44**
3	Functional impairment			-	0.61**	0.43**
4	Self-reported climate anxiety				-	0.41**
5	Generalized anxiety					-

**p < 0.001.

### 3.3 Sociodemographic influences on climate change anxiety

**Figure 1:**
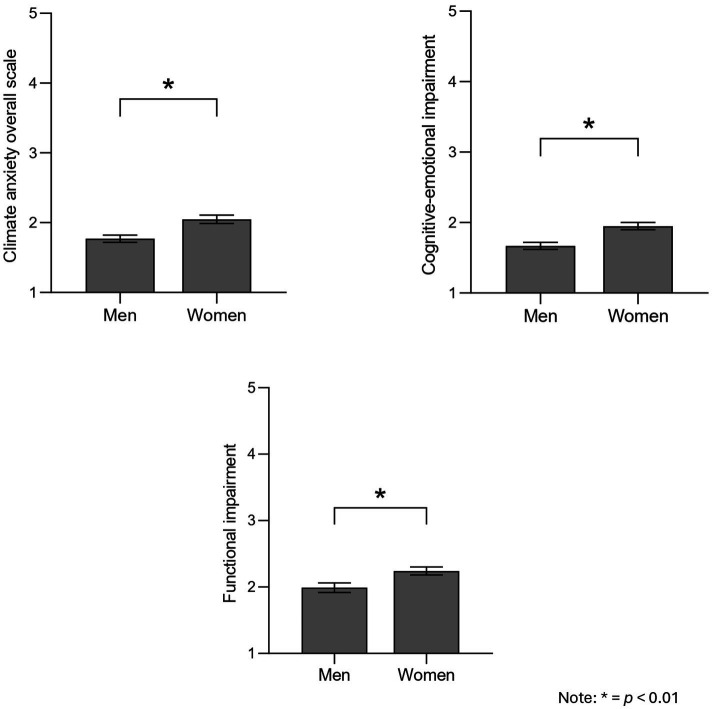
Gender differences in climate anxiety. *p < 0.01.

As seen in Figure 1, men and women differed in terms of climate change anxiety, *F* (1, 243) = 12.33, *p* < 0.001, ηp2 = 0.048, with women showing higher anxiety levels (*M* = 2.05, SE = 0.06) than men (*M* = 1.77, SE = 0.05). Further, analyses focusing on cognitive-emotional and functional impairment levels showed similar results, *F* (2, 241) = 6.63, *p* = 0.002; Pillai *V* = 0.05, ηp2 = 0.052. Follow-up univariate tests indicated that both cognitive-emotional, *F* (1, 243) = 13.32, *p* < 0.001, ηp2 = 0.052, and functional impairment, *F* (1, 243) = 6.98, *p* = 0.009, ηp2 = 0.028, differed by gender. Specifically, women showed higher cognitive-emotional and functional impairment (cognitive-emotional: *M* = 1.95, SE = 0.51; functional: *M* = 2.24, SE = 0.61) than men (cognitive-emotional: *M* = 1.67, SE = 0.05; functional: *M* = 1.99, SE = 0.07). This set of results fully confirms hypothesis H2.

**Figure 2:**
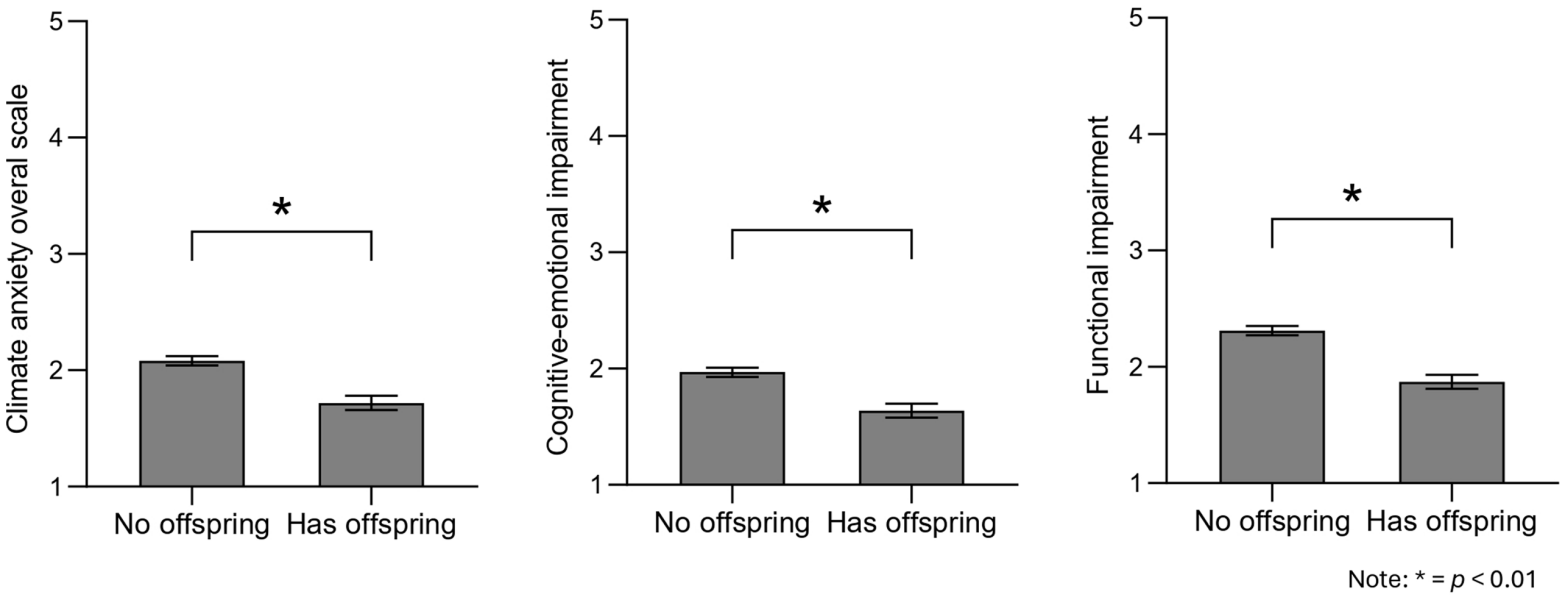
Differences in climate anxiety depending on parental status. *p < 0.01.

Regarding having or not having children, analyses showed differences, *F* (1, 243) = 21.81, *p* < 0.001, ηp2 = 0.081. People who do not have children (*M* = 2.08, SE = 0.04) experienced higher climate change anxiety than those who do (*M* = 1.72, SE = 0.06). Further, multivariate analyses on the specific subscales also showed that scores on the subscales varied depending on this: *F* (2, 246) = 11.78, *p* < 0.001; Pillai V = 0.05, ηp2 = 0.087. Again, follow-up univariate tests indicated that both impairment subscales differed based on whether people had children or not (cognitive-emotional: *F* (1, 248) = 16.91, *p* < 0.001, ηp2 = 0.064; functional: *F* (1, 243) = 22.59, *p* < 0.001, ηp2 = 0.084). Particularly, people who do not have children showed higher cognitive-emotional and functional impairment (cognitive-emotional: *M* = 1.97, SE = 0.04; functional: *M* = 2.31, SE = 0.05) than those who reported having children (cognitive-emotional: *M* = 1.64, SE = 0.06; functional: *M* = 1.87, SE = 0.07) (Figure 2). These results refute our H3.

Results also suggest that climate change anxiety and people’s age are related, as age was negatively and moderately associated with the scale, *r* (249) = −0.36, *p* < 0.001, as well as to its cognitive-emotional sub-scale, *r* (249) = −0.35, *p* < 0.001, and functional impairment sub-scale, *r* (249) = −0.32, *p* < 0.001. This corroborates our H4, indicating that older scholars present lower anxiety levels.

**Figure 3:**
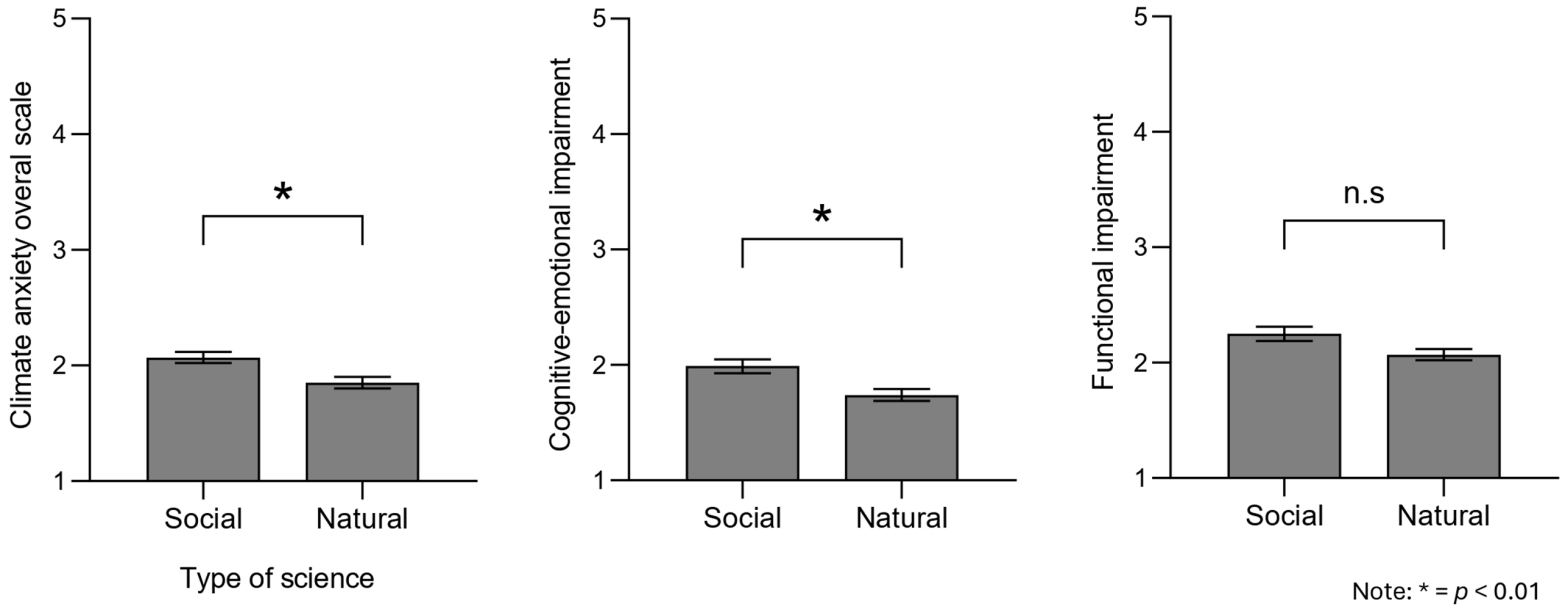
Differences in climate anxiety depending on field of study. *p < 0.01.

Our H5 was also confirmed since the results indicated that participants’ science field also mattered, *F* (1, 248) = 8.62, *p* = 0.004, ηp2 = 0.034; specifically, scientists working on social sciences showed higher climate change anxiety levels (*M* = 2.07, SE = 0.05) than scientists who work on natural sciences (*M* = 1.85, SE = 0.05). Further, multivariate analyses showed that impairment subscales also varied depending on the science field, *F* (2, 246) = 5.38, *p* = 0.005; Pillai V = 0.04, ηp2 = 0.042. However, in this case, follow-up univariate tests showed that even though cognitive-emotional impairment differed significantly by science field, *F* (1, 248) = 10.42, *p* = 0.001, ηp2 = 0.040, with social scientists (*M* = 1.99, SE = 0.06) scoring higher than natural scientists (*M* = 1.74, SE = 0.05), functional impairment did not, F (1, 248) = 3.70, *p* = 0.055, ηp2 = 0.015 (Figure 3).

**Figure 4:**
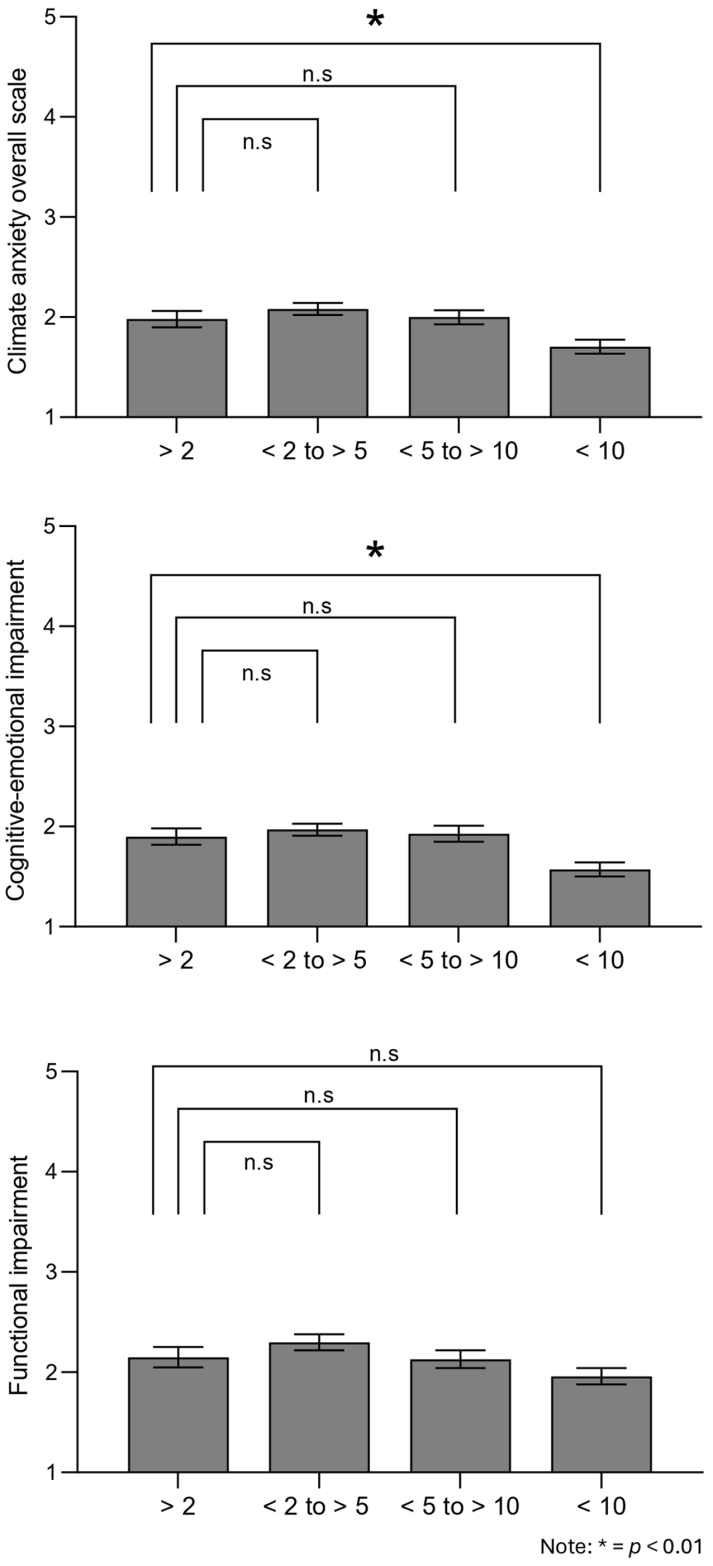
Differences in climate anxiety depending how long people have been working on climate change-related topics. *p < 0.01.

Lastly, results show that, even though overall levels of climate anxiety varied depending on how many years participants had worked on these topics, *F* (3, 245) = 5.14, *p* = 0.002, ηp2 = 0.059. Post-hoc comparisons showed that participants that have been studying climate change for relatively little time (< 2 years; *M* = 1.98, SE = 0.08) reported climate anxiety levels that were significantly different only to those working in the field for a long time (> 10 years; *M* = 1.70, SE = 0.07, *p* = 0.014). People working on climate change for more than ten years showed lower climate anxiety than those that are just starting. Similarly, analyses indicated that cognitive-emotional and functional impairment levels varied based on how long participants have been working on these topics, F (6, 490) = 3.51, *p* = 0.002; Pillai V = 0.08, ηp2 = 0.041, and follow-up univariate tests suggested that both the cognitive-emotional factor of climate anxiety, F (3, 245) = 6.07, *p* < 0.001, ηp2 = 0.069, and functional impairment one, *F* (3, 245) = 2.69, *p* = 0.047, ηp2 = 0.032, showed differences. However, a closer inspection to the post-hoc comparisons revealed that the difference between participants that were just starting to work on these topics (< 2 years; *M* = 1.90, SE = 0.08) and those that have been working for more than ten years (*M* = 1.57, SE = 0.07, *p* = 0.005) appeared only when focusing on the cognitive-emotional impairment factor. The functional impairment factor of climate anxiety did not show any difference based on how long people have worked on climate change (all ps > 0.05) (Figure 4). All in all, H6 was partially supported.

## 4 Discussion

To an extent, most Chilean climate change scientists are being emotionally affected by climate change. However, relatively high levels of self-reported climate change anxiety (73.1%) contrast with the moderate results obtained via the use of our adapted version of the Clayton and Karazsia’s scale, and in each of its subscales, cognitive-emotional and functional. Thus, this sample of Chilean scholars is not reporting excessively high levels of general climate change anxiety. While for most participants climate change is triggering some psychological distress, the emotional and practical consequences of this are still relatively mild. Yet, significant differences were found within the sample showing that women, young people, and those who do not have children, express more emotional and functional impacts associated with climate change. These impacts can manifest in various ways, including sleep disturbances, impaired concentration, difficulties managing climate change-related emotions, questioning the validity of personal emotional responses, reduced enjoyment of social activities, impaired ability to complete tasks, and persistent overthinking. Ultimately, these challenges can significantly impact both scholars’ work and their broader social lives.

Moreover, social scientists showed higher climate change anxiety levels than natural scientists. Overall, these results mirror findings observed in the general population, especially regarding gender and age influences (Gago and Sá, 2021; Hickman et al., 2021; Vaznonienė, 2023), and contribute to the current debates on the associations between climate anxiety and reproductive decisions especially among those who do not have children.

We propose some explanations for these results that could be tested in future studies, considering the exploratory character of this research. On the one hand, it is noticed that climate change anxiety might function in a similar way to other anxiety-related issues. Climate change could be a chronic concern, something that might even be in the background of people’s daily lives, but the extent to which this can interfere with scholar’s work, social life, and other dimensions varies depending on multiple factors. For example, the lower impacts on older people and natural scientists might suggests that those working with climate change for a longer period could have develop more or better coping mechanisms (Pihkala, 2020; Sapiains et al., 2015) to deal with such negative information. This may involve an increased sense of self-efficacy (Innocenti et al., 2023) linked to participation in science-policy interface initiatives, such as engagement in public committees or panels (where senior scientists are more likely to be invited to participate). Additionally, it could reflect a feeling of being part of a broader scientific community committed to collective action, which can enhance a sense of agency in addressing climate change challenges (Schwartz et al., 2023). However, these results might also represent coping mechanisms, where scientists question the societal role of their participation, potentially moderating their sense of responsibility—particularly when influenced by traditional views of science as mainly a generator of knowledge (Innocenti et al., 2023; Schwartz et al., 2023).

Disciplinary differences, one of the main findings of our study, might be associated with the fact that natural scientists are more likely to have a comprehensive knowledge of nature or changes of similar magnitude to those being experienced due to the ongoing climate change. For instance, several large extinctions of mammals and other animals have occurred in the past mediated through the release of greenhouse gases to the atmosphere, although triggered by natural processes and over a much longer period than current climate change (for instance during the Paleocene-Eocene thermal maximum 58 million years ago) (Keller et al., 2018). Early exposure of natural scientists to information about similar events during their training could provide a certain protection to anxiety to the ongoing climate change as they are more familiar to the occurrence of large magnitude changes on Earth. Future studies could identify such coping mechanisms to improve how these issues can be addressed in mental health interventions with the general population. On the other hand, it might also be the case that emotional expressions are more socially accepted among social scientists (especially those related to psychology or other mental health disciplines) than among natural scientists, which could prevent or make difficult for some researchers to recognize this type of psychological affectation. This could also apply to the young and female participants who more frequently expressed their negative emotions. In fact, in western societies men are less likely to express emotions than women when facing negative situations, and when they do, some studies suggest that men tend to express mostly emotions related to anger and frustration while women tend to express emotions more related to sadness and fear (Lebel and Lebel, 2018; Rausch et al., 2008), which are more connected to anxiety.

Finally, our data mounts criticisms on the eco-moms theory (Hunter, 2011; Price and Bohon, 2019), as scholars without children expressed higher levels of climate anxiety than those with children. In this case, climate change might be one of the reasons why they decided not to have children, and for those who do have children, a motivation to be more optimistic or at least to develop better coping mechanisms. Considering the young age of our sample, it is interesting to note that the results of a recent study in Chile indicate that 2 out of 3 young people who declare not wanting to have children express that climate change has influenced that decision (Instituto Nacional de la Juventud, 2024). On the other hand, it should also be considered that Chile has a low fertility rate of around 1.3 children per woman, with a continuing tendency to postpone motherhood until after age 30 (Instituto Nacional de Estadísticas (INE), 2024). Thus, this group of young scientists may have several reasons for not having children but climate change is likely one of them.

### 4.1 Limitations and future research recommendations

Our study is not exempt of limitations. First, while this work is, to our knowledge, the only one that has evaluated whether Chilean scholars working on climate change-related topics are experiencing climate anxiety, its cross-sectional nature only allowed us to examine this issue at a single point in time. As such, we cannot make causal inferences based on our results, for example, whether the number of years working on climate-related issues causally impacts climate anxiety. Instead, our findings may reflect age cohort differences. Future research could adopt a longitudinal approach to explore whether this psychological affectation changes over time and whether the predictors of climate anxiety evolve as well. A study by Chan et al. (2024) shows an example looking at the bi-directional associations of general and climate change anxiety over time (Chan et al., 2024).

Second, although focusing on a country like Chile to examine how frequent exposure to climate change information due to working on climate change research affects climate anxiety seems theoretically and contextually appropriate, given that Chile is and will continue to be heavily impacted by climate change, this focus limits the generalizability of our findings. Future studies should examine whether our results replicate in countries where the expected impacts of climate change differ and where the social and political contexts surrounding the issue are not the same as those in a Global South country.

Finally, it is worth noting that, as with most psychological conditions, climate change anxiety and its effects on daily functioning are likely influenced by a range of factors, including prior and/or concurrent psychological difficulties and the availability of social support. Exploring how these and other health- and socially-related factors might moderate our findings was beyond the scope of the present study, and no data were collected on these aspects. Indeed, a recent review of the literature on the psychological outcomes of climate change suggests that factors such as limited health infrastructure, along with the challenging economic and social conditions commonly found in Latin American countries, tend to exacerbate the negative psychological impacts of climate change (Palomo-Vélez et al., 2025). As such, future research should build on these results by explicitly modeling these potentially associated factors or by controlling for them as potential confounders.

## 5 Conclusion

Although for most participants climate change anxiety is not affecting life functioning, this does not necessarily mean that it will not affect them in the future, considering that climate change impacts, including extreme weather events, are likely to increase in the coming years and the associated consequences are also likely to be more complicated to be addressed if political, economic and social issues facing the country are not solved, all of which could increase climate anxiety in both the general population and the scientific community. More urgent, climate anxiety is already affecting some scholars, especially women, young, with no children and from social sciences. This anxiety can significantly impact their cognitive processes, emotional states, and functional abilities, with subsequent consequences for their professional and social lives.

We strongly believe that research centres and teams must develop strategies to help scholars cope with the psychological consequences of working on climate change. In Chile, and probably in many Latin-American countries, this is particularly important, considering the number of scholars studying climate change will likely keep growing, whereby more people, especially young will be working on this issue and probably being continuously exposed to climate data and experiencing climate change impacts. Such initiatives should provide safe spaces for researchers to express their emotional responses to climate change and could also aim at the development of coping mechanisms to increase self-efficacy through collective action. Thus, this study could be used as a baseline to monitor changes in the coming years, assessing the prevalence of climate change anxiety and mental health-related issues, and the impacts of future mental health initiatives in the scientific community that could also use these results. Future studies might collect new data to compare these results with, for instance, people who work in research centers in non-researcher positions, or with undergraduate students. Finally, although this was not our focus, this study could contribute to the ongoing discussion about different ways to assess climate anxiety and eco-anxiety in Latin America as showed in recent publications (Mejia et al., 2024; Rodríguez Quiroga et al., 2024).

## Data Availability

The datasets presented in this article are not readily available because it is confidential information, but we are keen to share it for scientific collaboration. Requests to access the datasets should be directed to rodolfo.sapiains@gmail.com.

## Footnotes

^1^Four participants chose ‘other’ and one participant chose ‘prefer not to say’ when asked about their gender. To meet the criteria for analysing group differences by sex, these participants were excluded from these analyses.
