# Supplementary material for: Climate change anxiety in the scientific community: an exploratory study with Chilean climate change-related scholars
Source: Front Psychol. 2025 Aug 13;16:1507487. doi: 10.3389/fpsyg.2025.1507487 (PMC12382390; doi:10.3389/fpsyg.2025.1507487)
Supplement: Supplementary file 1 [file Supplementary_file_1.docx]

| **Annex 1.** Verbatim text of measured items | | |
| --- | --- | --- |
| Subscale | Original item | Spanish version |
| Cognitive-emotional impairment | Thinking about climate change makes it difficult for me to concentrate | Pensar en el cambio climático dificulta mi capacidad de concentración |
|  | Thinking about climate change makes it difficult for me to sleep | Pensar en el cambio climático me dificulta dormir |
|  | I have nightmares about climate change | Tengo pesadillas sobre el cambio climático |
|  | I find myself crying because of climate change | Pensar en el cambio climático me da ganas de llorar |
|  | I think, “why can't I handle climate change better?” | Siento que no puedo manejar mis emociones frente al cambio climático |
|  | I go away by myself and think about why I feel this way about climate change | Cuestiono por qué me siento tan mal por el cambio climático |
|  | I write down my thoughts about climate change and analyze them | Escribo mis pensamientos sobre el cambio climático y los analizo |
|  | I think, “why do I react to climate change this way?” | Me cuestiono por qué reacciono tan mal frente a noticias sobre cambio climático |
| Functional impairment | My concerns about climate change make it hard for me to have fun with my family or friends. | Mis preocupaciones sobre el cambio climático hacen que sea difícil para mí divertirme con mi familia o amigo/as |
|  | I have problems balancing my concerns about sustainability with the needs of my family. | Tengo problemas para equilibrar mis preocupaciones sobre el cambio climático con las necesidades de mi familia |
|  | My concerns about climate change interfere with my ability to get work or school assignments done | Mis preocupaciones sobre el cambio climático afectan negativamente mi capacidad para trabajar y/o estudiar |
|  | My concerns about climate change undermine my ability to work to my potential. | Not measured |
|  | My friends say I think about climate change too much | Mis amigo/as dicen que pienso demasiado en el cambio climático |
| Experience | I have been directly affected by climate change | El cambio climático me afecta directamente |
|  | I know someone who has been directly affected by climate change | Not measured |
|  | I have noticed a change in a place that is important to me due to climate change | He percibido cambios negativos en lugares importantes para mí debido al cambio climático |
